# Editorial for Special Issue—‘’Research Progress and Applications of Natural Products”

**DOI:** 10.3390/molecules28145449

**Published:** 2023-07-17

**Authors:** Claudiu N. Lungu, Ionel Mangalagiu

**Affiliations:** 1 Department of Morphological and Functional Science, University of Medicine and Pharmacy, Dunarea de Jos, 800017 Galati, Romania; 2 Department of Chemistry, Alexandru Ioan Cuza University of Iasi, 11 Carol 1st Bvd., 700506 Iasi, Romania; ionelm@uaic.ro

This Special Issue (S.I.) focuses on natural products (N.P.s) as an inspiring resource for new bioactive molecules. Biologically active molecules have been discovered due to technological advances in medicinal chemistry and N.P. research [1]. The N.P.-based molecules discovered are derivatized using experimental methods to accelerate the process of drug discovery. The N.P.s and their primary and secondary metabolites are excellent starting points. Billions of structures are stored in vast databases and explored using experimental methods [2].

This S.I. focuses on the antimicrobial, antioxidant, antiphotoaging, and anti-inflammatory properties of N.P.s. 

Antimicrobial activity is described in the context of homodrimane sesquiterpenoids with a benzimidazole unit. A series of N-homodrimenoyl-2-amino-1,3-benzimidazoles and 2-homodrimenyl-1,3-benzimidazoles were studied. These compounds showed promissing biological activity (compared to the standard) for fungi—*Aspergillus niger*, *Fusarium solani*, *Penicillium chrysogenum*, *P. frequentans*, *Alternaria alternata*—and bacteria—*Bacillus* sp. and *Pseudomonas aeruginosa* [3]. These compounds can presumably present alternative treatments, especially in cases of drug resistance to Bacillus and Pseudomonas strains [4,5].

Antioxidant abilities and antibacterial activities are observed in 8,9-dihydro cannabidiol-based compounds. These compounds are viewed as viable alternatives to cannabidiol. In one study, 8,9-dihydro cannabidiol (H2CBD) was synthesized, and its structure–function relationship was studied. The H2CBD presented a much stronger antibacterial activity than the popular antibiotics assayed. The results suggest that H2CBD can be a valuable alternative to CBD. These compounds have high levels of bioactivity with respect to their antibacterial, bactericidal, and antioxidant activities. Their lower levels of toxicity to human skin fibroblasts makes them valuable drug candidates [6].

Another topic addressed in this S.I. is the anti-photoaging effect of *Phaseolus angularis*. Its antioxidant properties were determined with the help of 1,1-diphenyl-2-picrylhydrazyl (DPPH), 2,2′-azino-bis-(3-ethylbenzothiazoline)-6-sulfonic acid (ABTS) radical scavenging, and reactive oxygen species (R.O.S.) assays. A *P. angularis* seed extract (PASE) shows remarkable antioxidant activity. The PASE can be further developed as an essential ingredient in anti-aging products like other N.P.s of the Fabaceae family [7,8].

This S.I. contains some extensive reviews of N.P.s of diverse origins. Critical types of N.P. compounds are presented. Pyrrole-2-carboxaldehyde is isolated from various sources, β-(1-3)-D-glucans with β-(1-6) single glucose residues are isolated from insects of the Blattidae family, diterpenes are extracted from marine-derived fungi, amino-conjugated N.P.s are acquired from various sources, and monoterpene pyridine alkaloids and cyclopenta[c]pyridine derivatives are extracted from various sources; all are described as critical sources of new molecular motifs that are essential in novel drug development strategies [9,10]. 

One review presented in this S.I. shows that pyrrole-2-carboxaldehydes show potential for use in various physiological fields, including in the treatment of diabetes mellitus. These molecules can be used in academic, industrial, or medicinal chemistry [11]. 

In addition, some sources of N.P.s, such as insects, are not ignored in this S.I. Insect-derived N.P.s are unique in their structures and great activities. Furthermore, this type of N.P. is widely used to expand the chemical space. A review presented in this S.I. provides meaningful and valuable relevant information for researchers, promoting the further development of lead compounds [12].

Marine-derived fungi are crucial resources of novel compounds that are widely used by the pharmaceutical industry to expand its compound libraries. In a review in this S.I., 237 diterpenes are summarized. Derived from fungi, the compound with the most significant cytotoxicity is conidiogenone C, and the compound with the most promising antimicrobial activity is, notably, aspewentin D [13,14,15]. 

While proteins are essential macronutrients, a review in this S.I. demonstrates that several amino acid–natural compound conjugates have optimal absorption–distribution–metabolism–excretion (ADME) properties that can be further used for conjugating other desirable compounds. Remarkably, a conjugate of piperine and valine revealed a high level of efficiency against amastigotes [16,17].

Monoterpene pyridine alkaloids (MPTAs) or cyclopenta[c]pyridines have shown potential antibacterial, insecticidal, antiviral, and anti-inflammatory activities. Like other N.P.s, motifs are a vast source of chemical exploration in areas related to therapeutic chemistry=. The further exploration of MTPAs and cyclopenta[c]pyridines in chemical and drug contexts is to be expected [18].

The research articles and reviews presented in this SI offer distinct perspectives regarding some crucial N.P.s. Furthermore, the manuscripts within this S.I. offer a vast amount of N.P. chemical formulas of N.P.s that can be further used computationally or experimentally to create compound libraries the in search for new drugs.

## References

[1] Medina-Franco J.L., Chávez-Hernández A.L., López-López E., Saldívar-González F.I. (2022). Chemical Multiverse: An Expanded View of Chemical Space. Mol. Inform..

[2] Awale M., Visini R., Probst D., Arús-Pous J., Reymond J.-L. (2017). Chemical Space: Big Data Challenge for Molecular Diversity. Chimia.

[3] Lungu L., Blaja S., Cucicova C., Ciocarlan A., Barba A., Kulcițki V., Shova S., Vornicu N., Geana E.-I., Mangalagiu I.I. (2023). Synthesis and Antimicrobial Activity Evaluation of Homodrimane Sesquiterpenoids with a Benzimidazole Unit. Molecules.

[4] Wash P., Batool A., Mulk S., Nazir S., Yasmin H., Mumtaz S., Alyemeni M.N., Kaushik P., Hassan M.N. (2020). Prevalence of Antimicrobial Resistance and Respective Genes among *Bacillus* spp., a Versatile Bio-Fungicide. Int. J. Environ Res. Public Health.

[5] Azam M.W., Khan A.U. (2018). Updates on the pathogenicity status of Pseudomonas aeruginosa. Drug Discov. Today.

[6] Wu Q., Guo M., Zou L., Wang Q., Xia Y. (2023). 8,9-Dihydrocannabidiol, an Alternative of Cannabidiol, Its Preparation, Antibac-terial and Antioxidant Ability. Molecules.

[7] Oh S., Zheng S., Fang M., Kim M., Bellere A.D., Jeong J., Yi T.-H. (2023). Anti-Photoaging Effect of *Phaseolus angularis* L. Extract on UVB-Exposed HaCaT Keratinocytes and Possibilities as Cosmetic Materials. Molecules.

[8] Prasetyo B.E., Rafika D., Laila L., Aminah F. (2019). Physical Evaluation and Anti-Aging Effect of Red Bean Ethanolic Extract (*Vigna angularis* (Wild.) Ohwi & Ohashi) Peel-Off Gel Mask. Open Access Maced. J. Med. Sci..

[9] Rodrigues T., Reker D., Schneider P., Schneider G. (2016). ChemInform Abstract: Counting on Natural Products for Drug Design. Cheminform.

[10] Tang S.W., Tang W.H. (2019). Opportunities in Novel Psychotropic Drug Design from Natural Compounds. Int. J. Neuropsychopharmacol..

[11] Matsugo S., Nakamura Y. (2023). Pyrrole-2-carboxaldehydes: Origins and Physiological Activities. Molecules.

[12] Liang S., Zhang Y., Li J., Yao S. (2022). Phytochemical Profiling, Isolation, and Pharmacological Applications of Bioactive Com-pounds from Insects of the Family Blattidae Together with Related Drug Development. Molecules.

[13] Qiu P., Xia J., Zhang H., Lin D., Shao Z. (2022). A Review of Diterpenes from Marine-Derived Fungi: 2009–2021. Molecules.

[14] Hou S.-H., Tu Y.-Q., Wang S.-H., Xi C.-C., Zhang F.-M., Wang S.-H., Li Y.-T., Liu L. (2016). Total Syntheses of the Tetracyclic Cyclopiane Diterpenes Conidiogenone, Conidiogenol, and Conidiogenone B. Angew Chem. Int. Ed. Engl..

[15] Li X.-D., Li X.-M., Li X., Xu G.-M., Liu Y., Wang B.-G. (2016). Aspewentins D-H, 20-Nor-isopimarane De-rivatives from the Deep Sea Sediment-Derived Fungus Aspergillus wentii SD-310. J. Nat. Prod..

[16] Arifian H., Maharani R., Megantara S., Gazzali A.M., Muchtaridi M. (2022). Amino-Acid-Conjugated Natural Compounds: Aims, Designs and Results. Molecules.

[17] Skwarecki A.S., Nowak M.G., Milewska M.J. (2021). Amino Acid and Peptide-Based Antiviral Agents. Chemmedchem.

[18] Zhang X., Tao F., Cui T., Luo C., Zhou Z., Huang Y., Tan L., Peng W., Wu C. (2022). Sources, Transformations, Syntheses, and Bioactivities of Monoterpene Pyridine Alkaloids and Cyclopenta[c]pyridine Derivatives. Molecules.

